# 2823. In Vitro Efficacy of Minocycline on Clinical Gram Negative Bacterial Isolates

**DOI:** 10.1093/ofid/ofad500.2434

**Published:** 2023-11-27

**Authors:** Omar M Abu Saleh, Patrick Crowley, Nischal Ranganath, Douglas Challener, Nicholas Streck

**Affiliations:** Mayo Clinic Rochester, Rochester, Minnesota; Mayo Clinic, Rochester, MN; Mayo Clinic, Rochester, MN; Mayo Clinic, Rochester, MN; Mayo Clinic, Rochester, MN

## Abstract

**Background:**

Urinary tract infections (UTIs) caused by multidrug-resistant Gram-negative bacilli (MDR-GNB) have become increasingly challenging to manage due to the limited number of available effective oral agents. Minocycline, a second-generation tetracycline with a broad antimicrobial spectrum and reasonable urinary concentration, has recently gained attention as a potential treatment option for MDR-GNB UTIs. In this study, we aim to review the in vitro minocycline susceptibility against a wide spectrum of urinary GNB isolates performed at our reference lab.

**Methods:**

We conducted a retrospective review of all minocycline susceptibility testing performed on urinary GNB isolates performed at the Mayo Clinic reference lab between 2013-2022. We stratified our results for different species based on phenotypic resistance profiles as well as known species-specific resistance association.

**Results:**

During the study timeframe, we identified 28,388 GNB isolates that underwent minocycline susceptibility testing. Among the Escherichia coli (E.Coli) isolates resistant to ceftriaxone, ciprofloxacin, or meropenem, minocycline susceptibility was noted to be 78%, 46%, 76%, respectively (Fig 1). Among the Klebsiella pneumonia (KP) isolate resistant to ceftriaxone, ciprofloxacin, or meropenem, minocycline susceptibilities were 47%, 40%, and 53% respectively (Fig 1). Among the high risk Amp-C group Citrobacter freundii, Enterobacter Cloacae and Klebsiella aerogenes, minocycline susceptibility rate was noted to be 87%, 77%, and 89%, respectively (Fig 2). Finally, among the isolate group of non-fermenters including Achromobacter spp, Acinetobacter spp & Stenotrophomonas spp, susceptibility rates were noted to be 65%, 94%, and 100%, respectively (Fig 3).

**Figure:**
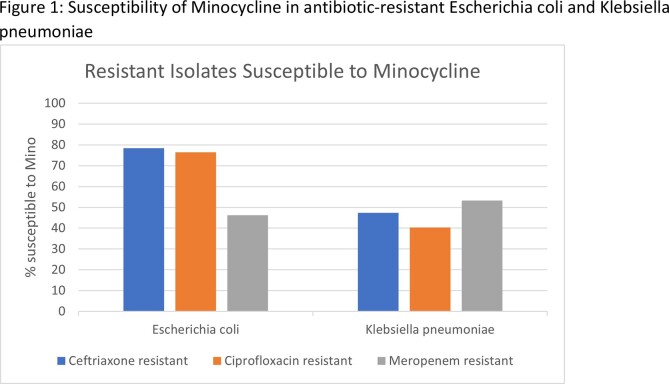

**Conclusion:**

Minocycline showed in vitro activity against many hard to treat GNB urinary isolates, including ceftriaxone and meropenem resistant E.coli & KP isolates. High-risk Amp-C producing GN species & some of the less common GN including Stenotrophomonas, Acinetobacter, and Achromobacter.

**Figure:**
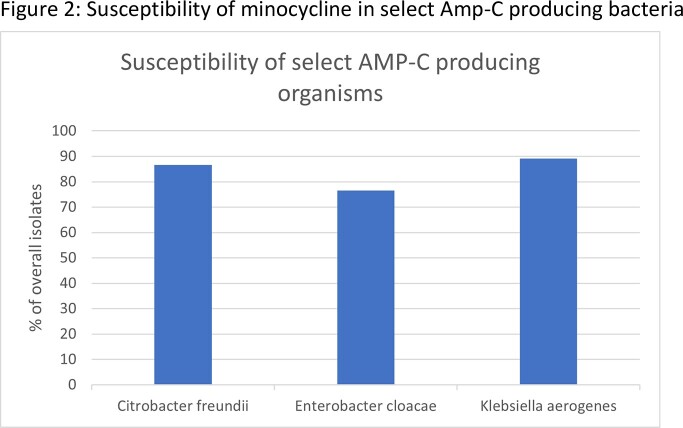

**Figure:**
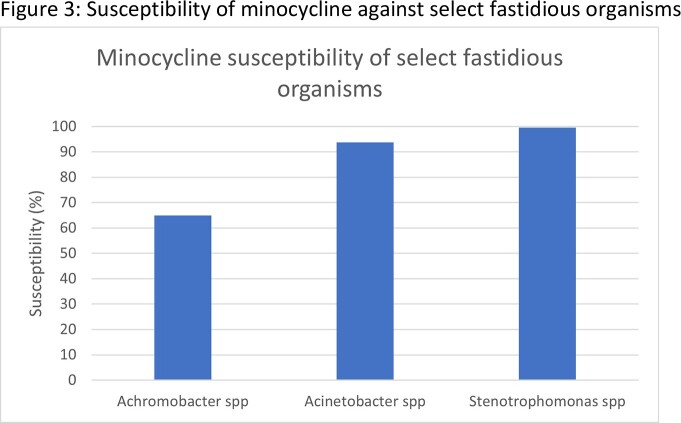

**Disclosures:**

**All Authors**: No reported disclosures

